# Classification of inherited neurometabolic disorders based on radiological aspects: pictorial essay

**DOI:** 10.1590/0100-3984.2021.0022

**Published:** 2022

**Authors:** André Felix Pedri, Marcelo dos Santos Guedes, Cláudio Campi de Castro

**Affiliations:** 1 Hospital Alvorada Moema, São Paulo, SP, Brazil.; 2 Hospital das Clínicas da Faculdade de Medicina da Universidade de São Paulo (HC-FMUSP), São Paulo, SP, Brazil.

**Keywords:** Metabolism, inborn errors/classification, Brain diseases, metabolic, inborn/classification, Diagnostic imaging/trends, Computed tomography, Magnetic resonance imaging, Erros inatos do metabolismo/classificação, Encefalopatias metabólicas congênitas/classificação, Diagnóstico por imagem/tendências, Tomografia computadorizada, Ressonância magnética

## Abstract

Inherited neurometabolic disorders represent a diagnostic challenge, and an efficient classification system is needed in order to improve the understanding of these diseases. Although they constitute a group of rare diseases, they have a collective incidence of at least one case per 1,000 live births. Some inherited neurometabolic disorders are treatable. The clinical and radiological presentations are variable and sometimes overlap, depending on the stage of the disease. Therefore, a number of classification systems have been devised, some of which are difficult to apply in practice. The aim of this study was to illustrate a classification system for inherited neurometabolic disorders, based exclusively on radiological findings. This was a retrospective study of imaging examinations of the central nervous system, particularly of children, performed in a network of hospitals. All of the cases were studied by multidetector computed tomography, magnetic resonance imaging, or both, the images having been obtained by two neuroradiologists. We included only cases in which a definitive diagnosis was made. The classification system separates the relevant radiological findings into 10 categories. All of the cases studied presented at least one of those findings. In most of the cases, more than one finding was observed, which increased specificity and narrowed the differential diagnosis. Data from the literature and from this study demonstrate that it is possible to classify inherited neurometabolic disorders by their radiological aspects, which favors a definitive diagnosis.

## Abstract (pt)

As doenças neurometabólicas hereditárias representam um desafio diagnóstico e precisam de classificações eficientes para sua compreensão. São um grupo de doenças raras, mas atingem pelo menos um a cada mil nascimentos. Algumas têm possibilidade terapêutica. Os quadros clínico e radiológico são variáveis e, às vezes, superponíveis, a depender da fase da doença. Por isso, observam-se na literatura vários tipos de classificações, algumas de difícil aplicação prática. O objetivo deste estudo é ilustrar a classificação das doenças neurometabólicas hereditárias baseada exclusivamente em achados radiológicos. Trata-se de um estudo retrospectivo a partir de exames de imagem do sistema nervoso central, particularmente de crianças, realizados em uma rede de hospitais. Todos os casos foram estudados por tomografia computadorizada multidetectores e/ou ressonância magnética, tendo sido avaliados por dois neurorradiologistas. Consideraram-se os casos com diagnóstico definitivo. A classificação propõe 10 subgrupos, de acordo com os achados radiológicos. Todos os casos estudados apresentaram, pelo menos, um desses achados. Na maioria dos casos observou-se mais de um achado, o que aumentou a especificidade e restringiu os diagnósticos diferenciais. Após avaliar os dados de literatura e os deste estudo, demonstra-se que é possível classificar as doenças neurometabólicas hereditárias pelo aspecto radiológico, o que favorece um diagnóstico definitivo.

## INTRODUCTION

Inherited neurometabolic disorders (INMDs) represent a diagnostic challenge. They constitute a group of rare and generally severe diseases. However, some INMDs are treatable. Despite some genetic variability, most INMDs are autosomal recessive disorders. Depending on the stage at diagnosis, the clinical and radiological presentations can be quite variable and overlapping, which makes the diagnostic process more complex^(1)^. Although various authors have proposed systems for classifying INMDs, some of those systems are difficult to apply^(1)^.

Technological advances in recent decades, especially in the field of imaging methods, have improved the sensitivity and specificity of the diagnosis of diseases of the central nervous system. Magnetic resonance imaging (MRI) and its advanced techniques play a central role in the study of INMDs^(1,2)^.

In view of the aspects described above, we identified the need to conduct a study based exclusively on the radiological findings of all INMDs, divided into subgroups for didactic purposes. Therefore, the objective of this study was to classify INMDs by their radiological aspects, in order to create a practical guide to be used as a reference.

This was a retrospective study based on data obtained from a network of hospitals. We reviewed multidetector computed tomography (MDCT) and MRI scans of the central nervous system of patients with INMDs, predominantly pediatric patients, who were evaluated between January 2010 and August 2020. Our analysis included only cases in which a definitive diagnosis was established on the basis of the anatomical, pathological, biochemical, or genetic findings. Thus, we included 74 confirmed cases. Those cases were divided into subgroups by the radiological findings.

We identified distinct patterns of neuroradiological findings. That allowed us to divide the findings into the following 10 subgroups: macrocrania; cysts; site(s) of white matter involvement; contrast enhancement; calcifications; basal nuclei involvement; restricted diffusion on diffusion-weighted imaging (DWI); vascular abnormalities; cranial nerve involvement; and metabolic profile on proton magnetic resonance spectroscopy ( ^1^H-MRS).

## MACROCRANIA

**Figure 1:**
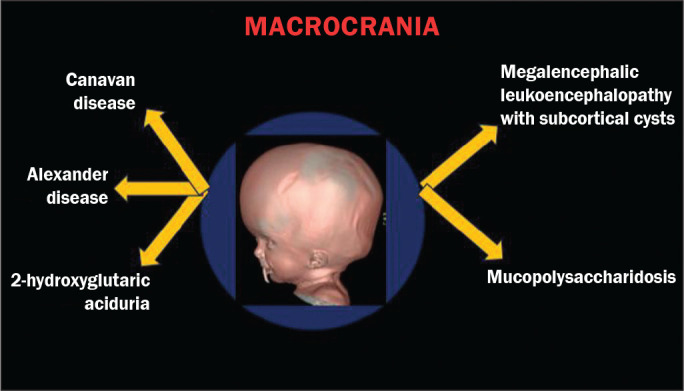
Macrocrania in INMDs. Three-dimensional volume rendering reconstruction, obtained from axial MDCT images of the skull, exemplifying macrocrania and its possible causes (arrows).

Macrocrania refers to increased head circumference. The INMDs that can present with macrocrania include megalencephalic leukoencephalopathy with subcortical cysts (MLC), Alexander disease, 2-hydroxyglutaric aciduria, mucopolysaccharidosis, and Canavan disease. Although macrocrania alone does not allow a definitive diagnosis to be made, it reduces the number of hypotheses and, when combined with other findings, can help define the diagnosis^(1-6)^, as depicted in Figure 1.

## CYSTS

**Figure 2:**
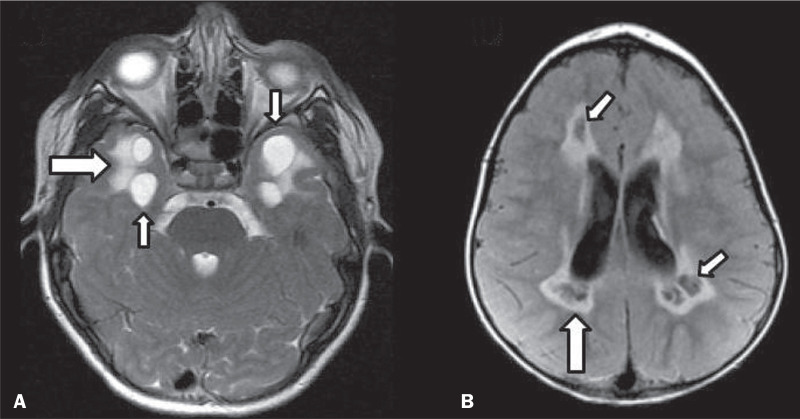
Presence of cysts in INMDs. MRI of the skull. A: T2-weighted axial TSE sequence in a patient with MLC, showing a hyperintense signal in the white matter, consistent with leukoencephalopathy (large arrow), containing subcortical cysts (small arrows) in the temporal regions. B: Axial FLAIR sequence in a patient with progressive cavitating leukoencephalopathy, showing a hyperintense signal, bilaterally, in the supratentorial periventricular white matter, consistent with leukoencephalopathy (larger arrow) and encompassing cysts (smaller arrows).

The imaging finding of a cyst can occur in a considerable proportion of individuals with an INMD^(1,2)^. The origin of such cysts remains unclear, although, in most cases, they must be associated with white matter degeneration, enlargement of the peri-encephalic spaces, or dilation of perivascular spaces. The location, number, and size of the cysts can facilitate the differential diagnosis. As illustrated in Figure 2, the following are the most common INMDs that can present with cysts^(1-6)^: MLC (subcortical cysts, predominantly in the temporal poles); glutaric aciduria type 1 (enlargement of the sylvian fissures); leukodystrophy with cysts and calcifications (Labrune syndrome); mucopolysaccharidosis (dilation of the perivascular spaces); progressive multifocal leukoencephalopathy (periventricular cysts and involvement of the corpus callosum); leukoencephalopathy with evanescent white matter (periventricular cysts); and Aicardi syndrome. Less common INMDs that can also present with cysts are as follows: Alexander disease (frontal cysts); Tay-Sachs disease; Zellweger syndrome (germinolytic cysts); leukoencephalopathy with brainstem and spinal cord involvement and lactate elevation (characterized by white matter lesions, together with tracts in the brainstem and posterior region of the spinal cord, which, due to the heterogeneity of the white matter signal, may include cyst-like images); Lowe syndrome; molybdenum cofactor deficiency; galactosemia, and Leigh syndrome (Figure 2).

## SITE OF WHITE MATTER INVOLVEMENT

**Figure 3:**
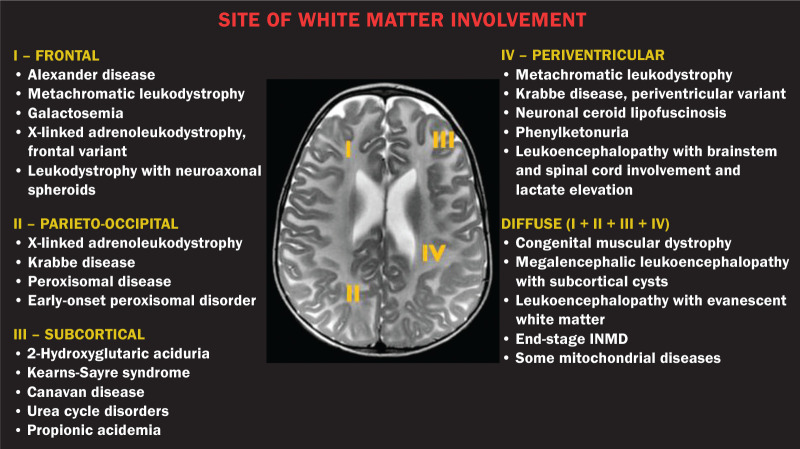
Schematic of white matter involvement and its possible causes in INMDs, based on a T2-weighted axial TSE MRI scan of the skull (regions labeled I to IV).

**Figure 4:**
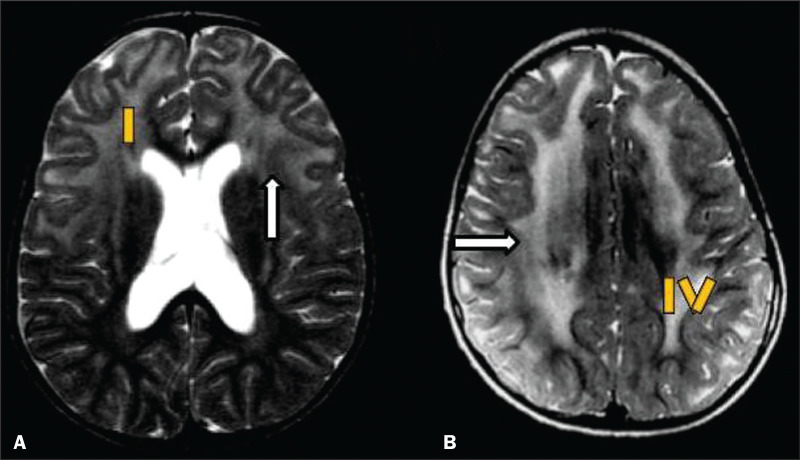
White matter involvement in INMDs. Axial MRI of the skull, correlated with the region numbering scheme in Figure 3. A: T2-weighted TSE sequence in a patient with Alexander disease, showing confluent hyperintense lesions in the frontal white matter (arrow and number I). B: T2-weighted TSE sequence in a patient with MLD, showing hyperintense signal intensity, bilaterally, in the upper periventricular white matter with a “tigroid” pattern (arrow and number IV).

One of the most significant radiological parameters in INMDs is the site of white matter involvement. In almost all of these diseases, the white matter involvement is typically in the supratentorial region. There may also be involvement of the brainstem and cerebellum^(1,2)^. As shown in Figure 3, the involvement can be confluent, multifocal, periventricular, subcortical, or diffuse, as well as being anterior, posterior, or both, although it is usually bilateral^(1,2)^. However, that there are variations and, depending on the stage of the disease at the time of the study, the assessment of white matter involvement may be hampered by the tendency of INMDs to present overlapping radiological signs in their chronic or end stage. The radiological finding known as a “tigroid” or “leopard skin” pattern (Figure 4), resulting from demyelination with preservation of some fibers in the perivenular spaces, is observed in metachromatic leukodystrophy (MLD) and in some individuals with globoid cell leukodystrophy (Krabbe disease) or leukoencephalopathy with evanescent white matter^(1-3)^.

## CONTRAST ENHANCEMENT

**Figure 5:**
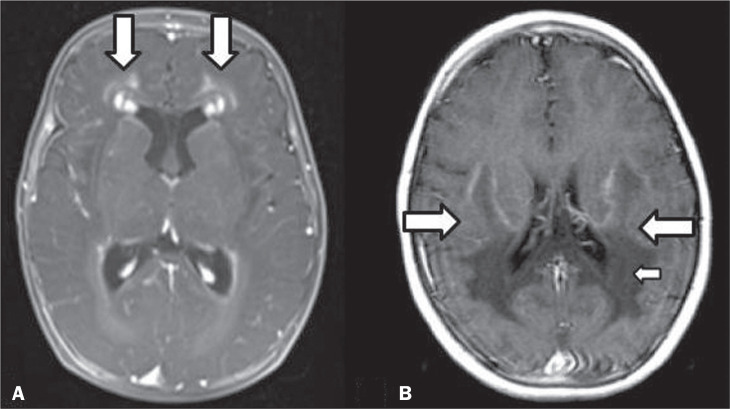
Contrast enhancement in INMDs. Contrast-enhanced MRI of the skull. A: T1-weighted axial spin-echo sequence in a patient with Alexander disease, showing “nodular” enhancement in the anterior periventricular region, forming the inverted V (“rabbit ear”) sign (arrows). B: T1-weighted axial spin-echo sequence in a patient with X-ALD, showing a hypointense signal and peripheral contrast enhancement of the parieto-occipital white matter, bilaterally. Note the three Schaumburg zones: the peripheral (anterior) zone, in which vasogenic edema predominates; the intermediate zone, in which there is active demyelination with enhancement (large arrows); and the central zone, in which there is gliosis (small arrow).

Some INMDs show parenchymal contrast enhancement (Figure 5), particularly X-linked adrenoleukodystrophy (X-ALD), Alexander disease, progressive cavitating leukoencephalopathy, Krabbe disease, and Labrune syndrome. However, such enhancement is most characteristic of Alexander disease with anterior periventricular “nodular” enhancement, forming the inverted V (“rabbit ear”) sign, and X-ALD, in which peripheral enhancement is divided into three distinct zones, known as Schaumburg zones^(1,2,6,7)^.

## CALCIFICATIONS

**Figure 6:**
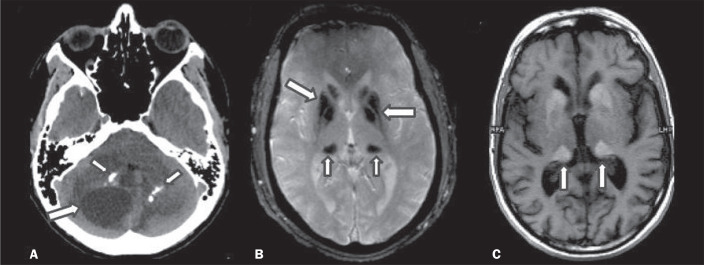
INMDs with calcifications. A: Unenhanced axial MDCT scan of the skull in a patient with Labrune syndrome, showing hypodense “cystic” images (large arrow), together with calcifications (small arrows), in the cerebellar hemispheres. B: Axial T2*-weighted MRI sequence in a patient with Fabry disease, showing hypointense foci, bilaterally, in the basal ganglia (large arrows) and in the pulvinar, forming the “pulvinar sign” (small arrows). C: Unenhanced T1-weighted axial spin-echo sequence in the same patient, showing a hyperintense signal in the same regions that showed a hypointense signal on the T2*-weighted sequence, including the pulvinar (arrows).

There are INMDs that present calcifications (Figure 6). This radiological finding is extremely relevant for the diagnosis of an INMD. Because of their high sensitivity and specificity, MDCT scans play an important role in the detection of such calcifications, the same being true for certain MRI sequences (T2*-weighted gradient-echo and susceptibility-weighted imaging). The INMDs that can present calcifications, predominantly in the basal ganglia, are Labrune syndrome, Aicardi syndrome, Fabry disease, mitochondrial encephalomyopathy, lactic acidosis, and stroke-like episodes (MELAS) syndrome, Kearns-Sayre syndrome, and, less commonly, Leigh syndrome. A diagnosis of X-ALD should also be considered when there are white matter calcifications^(1-3,7,8)^.

## INVOLVEMENT OF THE BASAL GANGLIA AND THALAMUS

**Figure 7:**
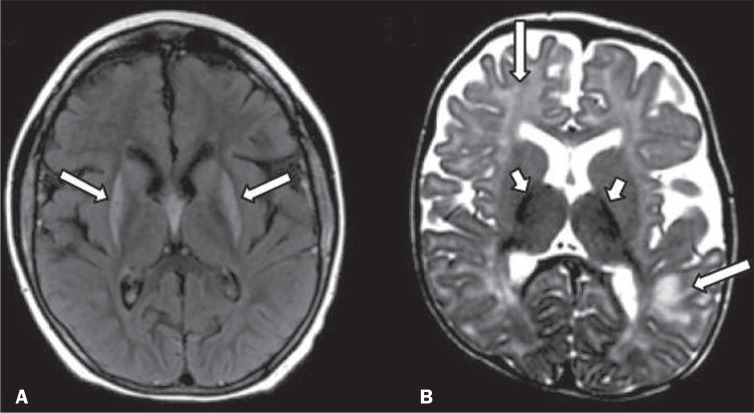
Involvement of the basal ganglia and thalamus in INMDs. MRI scans of the skull. A: Axial FLAIR sequence in a patient with Leigh syndrome, showing bilateral, symmetrical areas with a hyperintense signal in basal ganglia, particularly in the putamina, with local volume reduction (arrows). B: Axial T2-weighted TSE sequence in a patient with GM1 gangliosidosis, showing mild thickening of and a hypointense signal in the thalamus (small arrows), together with a diffuse hyperintense signal in the periventricular and subcortical white matter.

In individuals with an INMD, findings other than calcifications can be seen in the basal ganglia and thalamus, such findings including hyperdense areas on MDCT and, in some cases, a hyperintense signal on T1-weighted spin-echo MRI in the thalamus, in Krabbe disease, mucopolysaccharidosis, and Tay-Sachs disease. A hyperintense signal on T2-weighted and fluid-attenuated inversion recovery (FLAIR) MRI sequences, particularly in the caudate nuclei, globi pallidi, and putamina, may appear in glutaric aciduria type 1, Leigh syndrome, and Kearns-Sayre syndrome. In some INMDs, T2-weighted turbo spin-echo (TSE) MRI sequences show the so-called hypointense thalamus sign (Figure 7): neuronal ceroid lipofuscinosis; GM1 and GM2 gangliosidosis; fucosidosis; Wilson’s disease; and POLR3-related leukodystrophy. Atrophy of the caudate nuclei and putamina can be seen in Huntington’s disease and in the end stage of some INMDs^(1-3,8,9)^.

## RESTRICTED DIFFUSION

**Figure 8:**
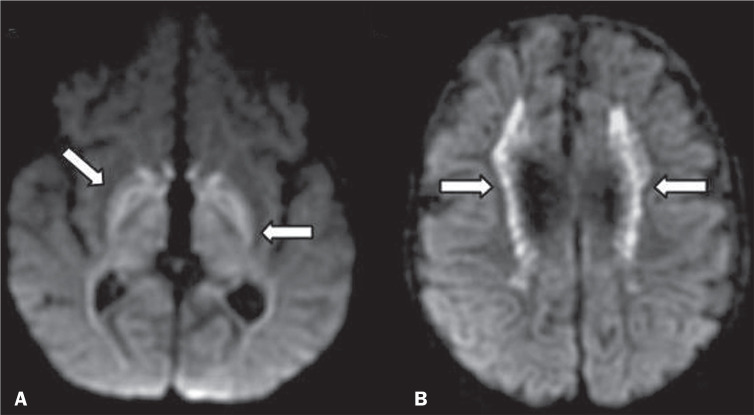
Restricted diffusion in INMDs. Axial acquisitions by DWI. A: Restricted diffusion in the basal ganglia and thalamus (arrows) of a neonate with maple syrup urine disease; nonketotic hyperglycinemia may have a similar radiological pattern. B: Bilateral restricted diffusion in the supratentorial periventricular white matter (arrows) of a patient with MLD.

Primarily in their initial or active stage, INMDs can present with parenchymal edema, which can be vasogenic, cytotoxic, or both. In cases of cytotoxic edema, DWI sequences may show restricted diffusion (Figure 8). The INMDs most commonly associated with restricted diffusion are as follows^(1,2,9,10)^: maple syrup urine disease; nonketotic hyperglycinemia; mitochondrial diseases, particularly Leigh syndrome and Kearns-Sayre syndrome; Krabbe disease; MLD; urea cycle disorders, progressive multifocal leukoencephalopathy; and, less frequently, glutaric aciduria type 1, phenylketonuria, and Canavan disease. It is also possible to perform diffusion tensor imaging to assess the microstructural involvement of the white matter in some INMDs^(11)^.

## VASCULAR ABNORMALITIES

**Figure 9:**
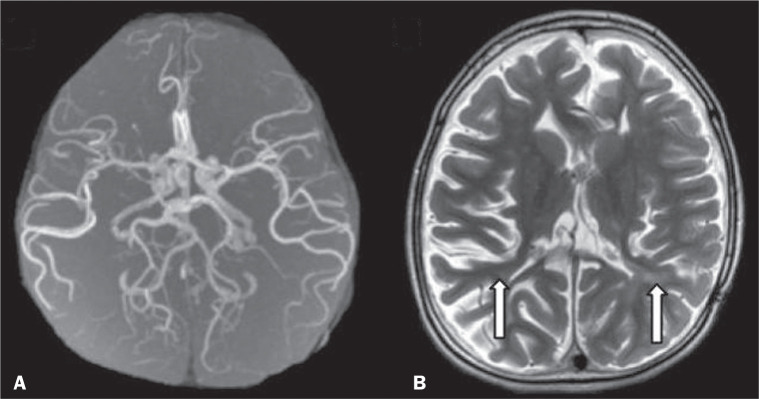
Vascular abnormalities in INMDs. MRI scans. A: Three-dimensional maximum-intensity-projection reconstruction of a three-dimensional time-of-flight magnetic resonance angiography scan, showing arterial elongation and tortuosity, in a child with Menkes disease. B: Axial T2-weighted TSE sequence in the same patient, showing cerebral atrophy accompanied by a slightly hyperintense signal in the posterior periventricular white matter, consistent with leukoencephalopathy (arrows).

There are INMDs that can result in vascular abnormalities, primarily Menkes disease, MELAS syndrome, Fabry disease, and mucopolysaccharidosis. Menkes disease is characterized by cerebral vascular tortuosity (Figure 9), whereas MELAS syndrome and Fabry disease are characterized by stroke and calcifications. In MELAS syndrome, there can also be a “shifting spread” (appearance, disappearance, and re-appearance in another region) of ischemic lesions at a vascular site within the parenchyma^(1-3,8)^.

## INVOLVEMENT OF THE CRANIAL NERVES

**Figure 10:**
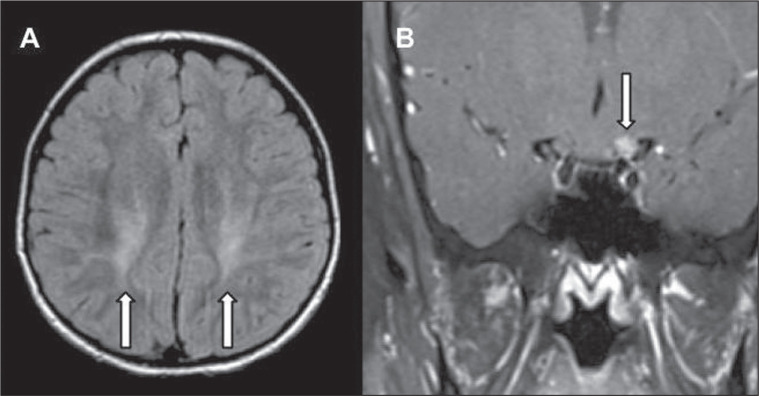
Cranial nerve involvement in INMDs. MRI scan. Axial T2-weighted FLAIR sequence (A) and contrast-enhanced coronal T1-weighted spin-echo sequence (B), in a patient with Krabbe disease, showing a hyperintense signal in the posterior periventricular white matter on the T2-weighted image (arrows in A), indicative of leukoencephalopathy, together with thickening and enhancement of the left optic nerve (arrow in B).

Cranial nerve involvement is uncommon in INMDs. When such involvement occurs, it can facilitate the differential diagnosis. Cranial nerve involvement can be seen in the following INMDs^(1,2,12)^: Krabbe disease (enhancement of the cranial nerves, particularly of the optic nerve; Figure 10); MLD (cranial nerve neuritis, excluding the optic nerve); and Leber hereditary optic neuropathy (enhancement of optical pathways).

## METABOLIC PROFILE/CHANGES ON ^1^H-MRS

**Figure 11:**
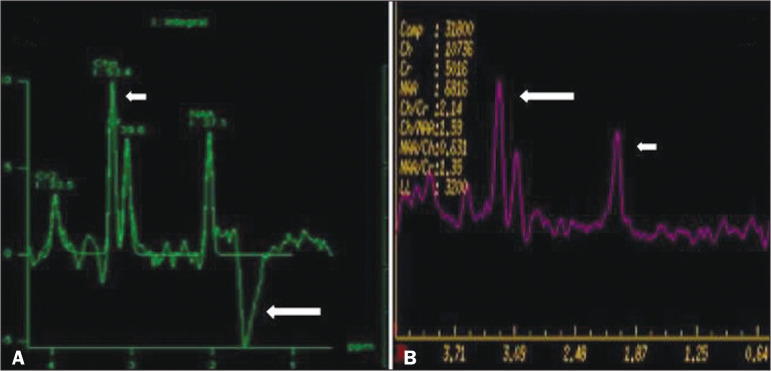
Changes on 1H-MRS in INMDs. A: Single-voxel spectroscopy image, with the voxel located in the lentiform nucleus, in a patient with mitochondrial disease, showing an inverted lactate peak (large arrow), below the baseline, with a TE of 135 ms, and a slightly elevated choline peak (small arrow). B: Single-voxel spectroscopy image, with the voxel located in the parietal white matter, showing an elevated choline peak (large arrow) and a below-normal N-acetyl-aspartate peak (small arrow), exemplifying a pattern that may be present in the chronic or end stage of most types of leukoencephalopathy, representing an nonspecific pattern.

Some INMDs can show changes on ^1^H-MRS. The ^1^H-MRS technique can employ sequences with a variety of echo times (TEs). Although the most commonly used TEs are 30 ms (short TE) and 130-144 ms (long TE), a TE of 270 ms is used in the evaluation of mitochondrial diseases. The most characteristic ^1^H-MRS alterations in INMDs are as follows: absence or reduction of the creatine peak (3.0 ppm, with short or long TE), in creatine deficiency; an elevated galactitol peak (3.7 ppm, with a short TE), in galactosemia; an elevated lactate peak (1.3 ppm, with traditional short and long TEs, as well as with a TE of up to 270 ms, with which the lactate can be observed as a double peak above the baseline), in mitochondrial diseases (Leigh syndrome, MELAS syndrome, Kearns-Sayre syndrome, etc.) in the clinical exacerbation stage and in leukoencephalopathy with brainstem and spinal cord involvement and lactate elevation (Figure 11A); an elevated N-acetyl-aspartate peak (2.0 ppm, preferably with a long TE), in Canavan disease; an elevated 2-hydroxyglutaric acid peak (2.5 ppm, with a short TE) in GA2; an elevated glycine peak (3.6 ppm, with a long TE, to differentiate glycine from myoinositol), in nonketotic hyperglycinemia; elevated glutamine/glutamate peaks (1.4-2.1 ppm, with short and long TEs), in urea cycle disorders; elevated peaks in ketoacid and lactate regions (0.9 ppm and 1.3 ppm, respectively, with short and long TEs, the latter being approximately 270 ms), in maple syrup urine disease; an elevated phenylalanine peak (7.37 ppm, with a short TE of approximately 20 ms), in phenylketonuria; and an elevated succinate peak (2.4 ppm, with a short TE), in Leigh syndrome. Krabbe disease, Zellweger syndrome, and MLD present a more nonspecific pattern, represented by an elevated choline peak, typically accompanied by a below-normal N-acetyl-aspartate peak (Figure 11B), a pattern that can also be seen in the chronic or end stage of other INMDs^(1,2,9,10)^.

## OTHER RELEVANT FINDINGS

There are other radiological findings that can facilitate the diagnosis of INMDs. Such findings include the following: malformations of cortical development associated with leukoencephalopathy, in Zellweger syndrome, glutaric aciduria type 1, and congenital muscular dystrophy; involvement of the brainstem, cerebellum, and spinal cord, sparing the supratentorial white matter, in juvenile or adult Alexander disease; subdural hematoma, in glutaric aciduria type 1, Menkes disease, and peroxisomal disease (in infants without Zellweger syndrome); bone lesions of the hands, ribs, and vertebral bodies, in mucopolysaccharidosis; and cerebellar involvement, together with thickening of the Achilles tendon, in cerebrotendinous xanthomatosis^(1,2)^.

## CONCLUSION

The diagnosis of INMDs constitutes a challenging branch of the field of neuroradiology, because many of the clinical and imaging aspects overlap between and among those disorders. However, by identifying the radiological findings suggested in the literature and those compiled in this essay, it is possible to narrow the differential diagnosis, bringing the diagnostic imaging process a step closer to the definitive diagnosis.
